# Replicate whole-genome next-generation sequencing data derived from Caucasian donor saliva samples

**DOI:** 10.1016/j.dib.2021.107349

**Published:** 2021-09-04

**Authors:** Marcus Høy Hansen, Charlotte Guldborg Nyvold

**Affiliations:** a Haematology-Pathology Research Laboratory, Research Unit for Haematology and Research Unit for Pathology, University of Southern Denmark and Odense University Hospital, Odense, Denmark; b Department of Hematology, Odense University Hospital, Odense, Denmark

**Keywords:** Whole-genome, Homo Sapiens genome, Next-generation sequencing (NGS), DNA sequencing, Raw data replicate

## Abstract

Next-generation sequencing (NGS) of whole genomes has become more accessible to biomedical researchers as the sequencing price continues to drop, and more laboratories have NGS facilities or have access to a core facility. However, the rapid and robust development of practical bioinformatics pipelines partly depends on convenient access to data for the testing of algorithms. Publicly available data sets constitute a part of this strategy.

Here, we provide a triplicate whole-genome paired-end sequencing data set, consisting of 1.38 billion raw sequencing reads derived from saliva DNA from a single anonymous male Caucasian donor, with the average sequencing depths aimed at 30x for two of the samples and 4x for a low-coverage sample. The raw number of single nucleotide variants were 3.3–4 million and the median variant read depth of GATK4-passed variants in three samples was 22, 18, and 10. 81% of all variants were found in two or three of the samples, whereas 19% were singletons. The karyotype was evaluated as 46,XY with no apparent copy-number variation.

*The data set is provided without restrictions for research, educational or commercial purposes.*

**Specifications Table**

**Table:** 

Subject	Health and medical sciences
Specific subject area	Human genome, triplicate raw control DNA sequence data for evaluation or educational purpose
Type of data	Triplicate paired-end DNA sequencing reads from Illumina Novaseq 6000 provided in compressed FastQ format (3 × 2 files available for download)
How data were acquired	See *data collection*
Data format	Raw gzipped sequencing data in the FASTQ format available in online repository.
Parameters for data collection	Not applicable
Description of data collection	Saliva was obtained from the donor, with approximately one year from the first sample to the third sampling. DNA purification and sequencing were performed in separate batches. Raw data is provided as obtained by the sequencing provider (Dante-Labs, L'Aquila, Italy)
Data source location	Haematology-Pathology Research Laboratory, Research Unit for Haematology and Research Unit for Pathology,University of Southern Denmark and Odense University Hospital, Odense, Denmark
Data accessibility	The data set is deposited in a public repository. Data are directly downloadable without access restrictions. Repository name: fig**share** (figshare.com) Data identification numbers: Replicate 1–3 Direct URL to data: https://doi.org/10.6084/m9.figshare.c.5336714

**Value of the Data**

• The data set provided here is relevant for the continued development and testing of bioinformatics pipelines as whole-genome sequencing become more important in biomedical research.
• Data access is provided by simple download and without restrictions. The triplicate sequencing of a Caucasian male may benefit bioinformaticians, biomedical researchers for testing or as control samples. The data may also be used for educational purposes.
• The raw sequencing data consists of biological replicates of low, medium, and higher coverage, which thus may be used for testing different workflow setups.

## 1 Data Description

**Fig. 1:**
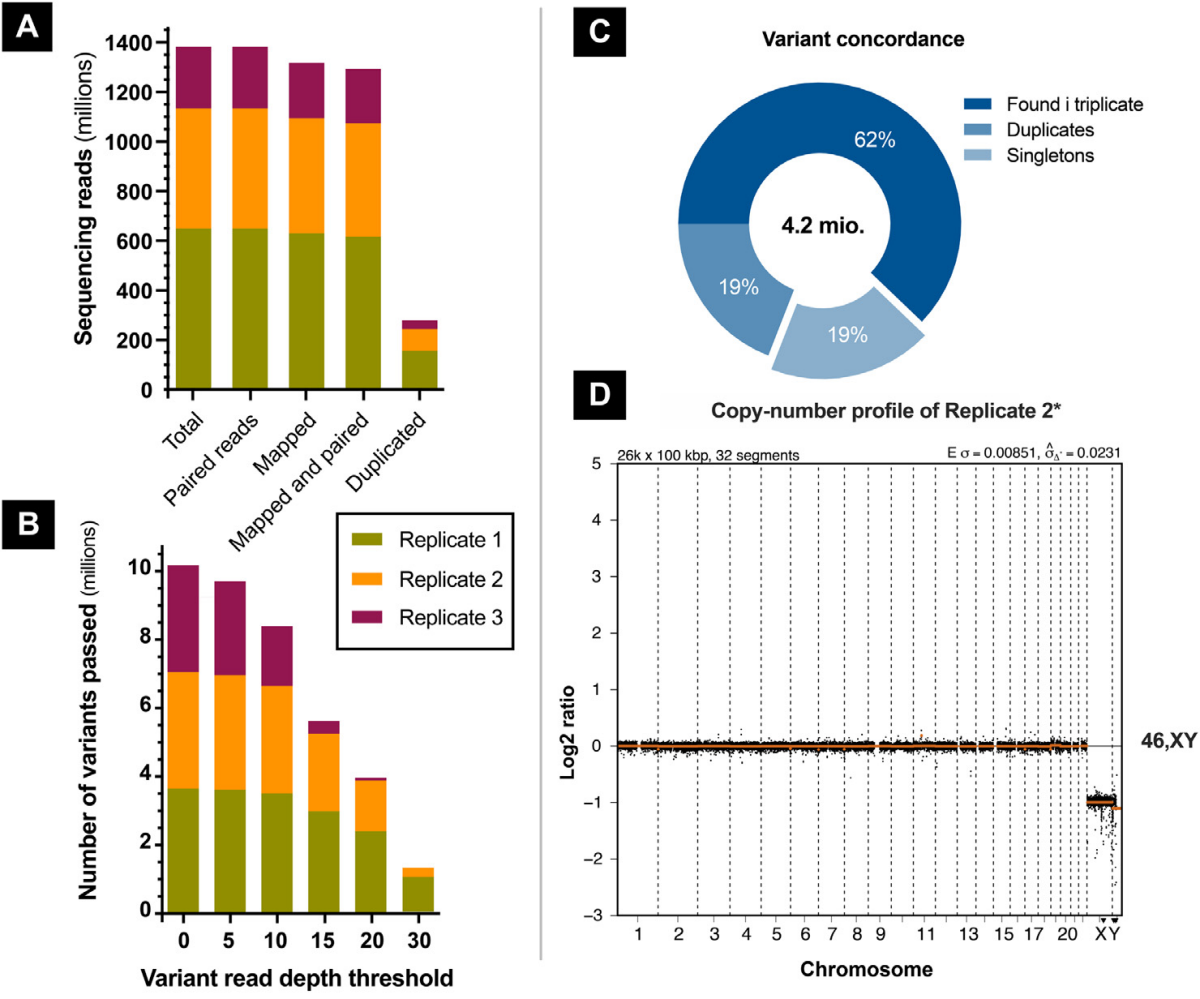
General statistics and quality assessment of the whole-genome sequencing replicates. The data set consists of 1.38 billion reads, in total, with a high fraction of reads being mappable (A). Each sample contained 3.3–4 million GATK-passed variants (B). 81% of the GATK-passed variants (4.2 million in total) were found in two or three samples, while 19% singletons were found (C). No apparent copy-number variation was detected in the samples (D, *Replicate 2* copy-number profile. *Sample with medium coverage is shown).

Here, we provide a data collection of samples derived from saliva DNA from a single anonymous male Caucasian donor consisting of triplicate whole-genome paired-end sequencing reads, with 1.38 billion raw reads in total (Fig. 1A), with a mean quality of 36 (SAMtools *stats*), and approximately 93.4% paired and mappable reads (GRCh37). The combined theoretical mean coverage was estimated to be 63–67x, depending on whether the unadjusted or mapped percentage was implemented, using the Lander and Watermann approach [1,2] for genomic mapping: *C* = L*N/G (C: coverage, *L*: read length, *N*: number of reads, *G*: genome size). Calculations were based on an average read length of 144, 146, and 148 bp. The median GATK-passed variant read depths of the three were 22, 18, and 10 with 3.3–4 million variants (Fig. 1B), thus representing medium, low and shallow depth in the perspective of contemporary WGS coverage. 81% of all GATK4-passed variants (see provided workflow), 4.2 million in total, were found in two or three of the samples and 19% in singletons (Fig. 1C). In agreement with previously reported results [3], the total number of unique single nucleotide variants were approximately 4 million. The karyotype was evaluated as 46,XY with no noticeable copy-number variation (CNV) detected (Fig. 1D). We note that the number of variants will vary according to user-specified workflow.

*The data set is provided without restrictions for research, educational or commercial purposes. Additional replicates may be added to the repository for future usage. Please cite appropriately.*

## 2 Experimental Design, Materials and Methods

Biological material was collected using GeneFiX Saliva DNA collection kits and stored at ambient temperature. DNA extraction, quality control, library preparation, and sequencing were performed by the sequencing provider (Dante-Labs, L'Aquila, Italy) over the time span of approximately one year from replicate 1 to 3. For the assessment shown here, alignment implemented Burrows-Wheeler Aligner [4] with the human reference genome GRCh38 and Genome Analysis Toolkit 4.1.9 [5]. Variant calling was based on deduplicated sequencing with the provided workflow. Quality was assessed with SAMtools stat/flagstat [6] and FastQC [7]. SNP comparisons were performed in the Wolfram (Mathematica, Wolfram Research, Ill, USA) and the CNV profile was assessed using QDNAseq [8] in R 3.6.1 using Ubuntu 18.04.

*Used commandline workflow*

fn=${1%_L001_R1_001.fastq.gz}
bwa mem -M -R ``@RG\tID:group1\tSM:$1\tPL:illumina\tLB:lib1\tPU:unit1'' -t 23 hg38/Homo_sapiens_assembly38.fasta $1 ${1%_L001_R1_001.fastq.gz}_L001_R1_001.fastq.gz | samtools view -@23 -m 1G -Sb -> ${1%_L001_R1_001.fastq.gz}.bam
gatk MarkDuplicatesSpark \
-I $fn.bam \
-O $fn.dedup.bam \
–remove-sequencing-duplicates
gatk BaseRecalibrator \
-I $fn.dedup.bam \
-R hg38/Homo_sapiens_assembly38.fasta \
–known-sites hg38/1000G_phase1.snps.high_confidence.hg38.vcf.gz \
–known-sites hg38/Homo_sapiens_assembly38.dbsnp138.vcf \
–known-sites hg38/1000G_phase1.snps.high_confidence.hg38.vcf.gz \
-O $fn.recal1.table
gatk ApplyBQSR \
-I $fn.dedup.bam \
-R hg38/Homo_sapiens_assembly38.fasta \
–bqsr-recal-file $fn.recal1.table \
-O $fn.dedup.recal.bam
gatk HaplotypeCaller \
–native-pair-hmm-threads 23 \
-R hg38/Homo_sapiens_assembly38.fasta \
-I $fn.dedup.recal.bam \
–dbsnp hg38/Homo_sapiens_assembly38.dbsnp138.vcf \
-O $fn.mnvs.vcf
gatk SelectVariants \
-R hg38/Homo_sapiens_assembly38.fasta \
-V $fn.mnvs.vcf \
–select-type-to-include SNP \
-O $fn.SNP.vcf
gatk VariantRecalibrator \
-R hg38/Homo_sapiens_assembly38.fasta \
-V $fn.SNP.vcf \
–resource:hapmap,known=false,training=true,truth=true,prior=15.0 hg38/hapmap_3.3.hg38.vcf \
–resource:omni,known=false,training=true,truth=true,prior=12.0 hg38/1000G_omni2.5.hg38.vcf \
–resource:1000G,known=false,training=true,truth=false,prior=10.0 hg38/1000G_phase1.snps.high_confidence.hg38.vcf \
–resource:dbsnp,known=true,training=false,truth=false,prior=2.0 hg38/Homo_sapiens_assembly38.dbsnp138.vcf \
-an QD -an MQ -an MQRankSum -an ReadPosRankSum -an FS -an SOR -an DP \
-mode SNP \
-O $fn.output.recal \
–tranches-file $fn.output.tranches \
–rscript-file $fn.output.plots.R
gatk ApplyVQSR \
-R hg38/Homo_sapiens_assembly38.fasta \
-V $fn.SNP.vcf \
-O $fn.SNP.filtered.vcf \
–truth-sensitivity-filter-level 99.5 \
–tranches-file $fn.output.tranches \
–recal-file $fn.output.recal \
-mode SNP

## Ethics Statement

Informed consent was obtained concerning the donation of biological material and genomic information. Sequencing was part of a technology assessment using anonymous donor material and does not involve any clinical evaluations or trials. Data is made freely available in order to contribute to the continued development of NGS bioinformatics and for educational purposes.

## CRediT Author Statement

**Marcus Høy Hansen:** Conceptualization, Methodology, Software, Validation, Formal analysis, Investigation, Resources, Data Curation, Writing, Editing, Visualization, Supervision, Project administration, Funding acquisition; **Charlotte Guldborg Nyvold:** Writing, Editing, Supervision.

## Declaration of Competing Interest

The authors declare that they have no known competing financial interests or personal relationships which have or could be perceived to have influenced the work reported in this article. Funding was provided by the first author. Disclaimer: The provided data presentation is deliberately descriptive. *It is not a regular scientific paper*.
